# Vaccines for the elderly: current use and future challenges

**DOI:** 10.1186/s12979-017-0107-2

**Published:** 2018-01-22

**Authors:** Birgit Weinberger

**Affiliations:** Universität Innsbruck, Institute for Biomedical Aging Research, Rennweg 10, 6020 Innsbruck, Austria

**Keywords:** Vaccine, Elderly, Aging, Immunosenescence, Influenza, Herpes zoster, *Streptococcus Pneumoniae*

## Abstract

Age-related changes of the immune system contribute to increased incidence and severity of infections in the elderly. Vaccination is the most effective measure to prevent infections and vaccination recommendations in most countries include specific guidelines for the elderly. Vaccination against influenza and *Streptococcus pneumoniae* is usually recommended for persons with underlying diseases and for the elderly with heterogeneous age limits between ≥ 50 years and ≥ 65 years. Some countries also recommend vaccination against herpes zoster. Several vaccines are recommended for all adults, such as regular booster shots against tetanus/diphtheria/pertussis/polio, or for specific groups, e.g. vaccination against tick-borne encephalitis in endemic areas or travel vaccines. These are also relevant for the elderly. Most currently used vaccines are less immunogenic and effective in the elderly compared to younger adults. Potential strategies to improve their immunogenicity include higher antigen dose, alternative routes of administration, and the use of adjuvants, which were all implemented for influenza vaccines, and induce moderately higher antibody concentrations. Research on universal vaccines against influenza and *S. pneumoniae* is ongoing in order to overcome the limitations of the current strain-specific vaccines. Respiratory syncytial virus causes significant morbidity in the elderly. Novel vaccines against this and other pathogens, for instance bacterial nosocomial infections, have tremendous potential impact on health in old age and are intensively studied by many academic and commercial organizations. In addition to novel vaccine developments, it is crucial to increase awareness for the importance of vaccination beyond the pediatric setting, as vaccination coverage is still far from optimal for the older population.

## Background

With increasing life expectancy, the global population ages, and the number of persons older than 60 years of age is expected to double by 2050, reaching 2.1 billion. The number of persons above 80 years is projected to increase even more dramatically from a worldwide total of 125 million in 2015 to 434 million in 2050 [1]. The severity of many infections is higher in the elderly compared to younger adults and infectious diseases are frequently associated with long-term sequelae such as impairments in activities of daily living, onset of frailty, or the loss of independence [2, 3]. This represents a serious challenge for public health systems, and the prevention of infectious disease is therefore an important measure to ensure healthy aging and improve the quality of life. The tremendous success of childhood vaccination is widely recognized, but the need for life-long vaccination programs and the importance of vaccination for the older population are frequently underestimated. This review summarizes current recommendations for developed countries, gives examples regarding immunogenicity and efficacy data for vaccines currently used for the elderly, and provides an outlook on novel vaccines developed specifically for this age group.

## Vaccines specifically recommended for the elderly

Many countries have established vaccination recommendations for adults and most of these also include specific guidelines for older adults. Vaccination against influenza and *Streptococcus pneumoniae* is usually recommended for persons with underlying diseases and for the elderly with heterogeneous age limits between ≥ 50 years and ≥65 years. Some countries also recommend vaccination against herpes zoster for older adults. Table 1 summarizes current recommendations for Europe and the USA [4, 5].

**Table 1:** Vaccination recommendations for older adults in Europe and the US

	Influenza^a^	S. pneumoniae^b^	Herpes zoster^d^	Tetanus	Diphtheria^i^	Pertussis^j^
Austria	all adults	> 50; PCV + PPV^c^	> 50	every 5 years^e^	every 5 years^e^	every 5 years^e^
Belgium	> 65	> 65; PCV + PPV^c^		every 10 years	every 10 years	once^h^
Bulgaria	> 65	–	–	every 10 years	every 10 years	–
Croatia	> 65	–		once at 60	–	–
Cyprus	> 65	> 65; PPV		every 10 years	every 10 years	–
Czech Rep.	all adults	> 65; PCV + PPV^c^	> 50	every 10 years^f^	–	once at 65
Denmark	> 65	> 65; PCV or PPV		–	–	–
Estonia	> 65	–		every 10 years	every 10 years	–
Finland	> 65	> 65; PCV or PPV		every 10 years	every 10 years	–
France	> 65	–	65-75	every 10 years^g^	every 10 years^g^	once^h^
Germany	> 60	> 60; PPV		every 10 years	every 10 years	once^h^
Greece	> 60	> 65; PCV	> 60	every 10 years	every 10 years	once^h^
Hungary	> 60	> 50; PPV		–	–	–
Iceland	> 60	> 60; PPV		–	–	–
Ireland	> 65	> 65; PPV		–	–	–
Italy	> 65	> 65; PCV + PPV^c^	> 65	every 10 years	every 10 years	every 10 years
Latvia	> 65	–		every 10 years	every 10 years	–
Liechtenstein	> 65	–		every 10 years^g^	every 10 years^g^	every 10 years^g^
Lithuania	> 65	–		every 5-10 years	every 5-10 years	–
Luxembourg	> 65	> 60; PCV + PPV^c^		every 10 years	every 10 years	every 10 years
Malta	all adults	> 65; PCV		–	–	–
Netherlands	> 60	–		–	–	–
Norway	> 65	> 65; PPV		–	–	–
Poland	all adults	> 50; PCV		once^h^	once^h^	–
Portugal	> 65	–		every 10 years^g^	every 10 years^g^	–
Romania	> 65	–		–	–	–
Slovakia	> 60	> 60; PCV		every 15 years	every 15 years	–
Slovenia	all adults	> 65;PCV or PPV		every 10 years	every 10 years	once^h^
Spain	> 65	> 65; PPV		once at 65	once at 65	–
Sweden	–	> 65; PPV		every 20 years	every 20 years	–
UK	> 65	> 65; PPV	> 70	–	–	–
USA	all adults	> 65; PCV + PPV^c^	> 60	every 10 years	every 10 years	once^h^

Shown are recommendations for the general population for all countries listed by the European Centre for Disease Prevention and Control. Specific recommendations for risk groups (co-morbidities, health care personnel etc.) are available in most countries

^a^annual vaccination, inactivated trivalent vaccine

^b^recommendation for persons without prior vaccination against pneumococcal disease; no booster shots recommended for general population; recommendations may differ for persons with underlying medical conditions

^c^one dose of PCV13 (pneumococcal conjugate vaccine) followed by 1 dose of PPV23 (pneumococcal polysaccharide vaccine)

^d^one dose, contraindicated in immunosuppressed patients (live vaccine)

^e^for persons over 65 years; every 10 years for younger adults

^f^for persons over 65 years; every 10-15 years for younger adults

^g^for persons over 65 years; every 20 years for younger adults

^h^once during adulthood

^i^vaccine containing reduced diphtheria dose (d)

^j^vaccine containing acellular pertussis antigens

### Influenza

Influenza causes approximately 100,000 hospitalizations and 36,000 deaths annually in the USA, which occur mainly in persons over the age 65 years [6, 7]. Vaccines against influenza usually contain three different strains (A/H1N1, A/H3N2, B) and the exact composition of the vaccine is determined each year by the World Health Organization (WHO) based on surveillance data. Recently, quadrivalent vaccines became available, as two different B strains had circulated in parallel for several years [8, 9]. Annual vaccination against influenza is recommended, as the composition of the vaccine changes in order to reflect currently circulating virus strains.

Immunogenicity of influenza vaccines is usually measured by hemagglutination inhibition assay (HAI), which quantifies antibodies specific for the viral hemagglutinin. Many studies demonstrated that antibody concentrations after vaccination are lower in older compared to younger adults [10] and that co-morbidities and frailty further decrease responsiveness to vaccination [11, 12]. Clinical efficacy or effectiveness of influenza vaccines is difficult to analyze, as compilations of results from clinical studies are highly complex. Parameters such as study population (age distribution, co-morbidities, frailty etc.), epidemiological factors (transmission patterns e.g. in institutionalized cohorts, prevalence of the virus), and virological factors (virulence, mismatch between vaccine and circulating viral strains) are different for each study and each influenza season. In addition, various clinical read-out parameters for influenza disease such as influenza-like illness (ILI), laboratory confirmed influenza or hospitalization due to influenza are utilized. Meta-analyses have estimated clinical benefits of influenza vaccination, and it can be concluded that protection is lower in older than in young adults [13, 14]. Several strategies to improve influenza vaccines for the elderly led to the licensure of vaccines containing the oil-in-water emulsion adjuvant MF59 [15] or 60 μg instead of 15 μg of hemagglutinin protein per dose [16], and of a vaccine administered via the intradermal instead of the intramuscular route [17]. These vaccines elicit slightly higher antibody responses compared to the standard inactivated vaccine. Interestingly, MF59-adjuvanted vaccines induce substantially higher antibody responses against heterologous vaccine strains [18, 19], and this broader neutralizing activity probably contributes to the higher clinical efficacy observed with the adjuvanted vaccine. A large trial in Italy demonstrated that the risk of hospitalization for influenza or pneumonia was 25% lower for the adjuvanted vaccine compared to non-adjuvanted vaccine (relative risk 0.75, 95% CI 0.57-0.98) [20]. In a study including residents of long-term care facilities, the risk of ILI was higher in persons receiving standard TIV (OR 1.52, 95% CI 1.22-1.88) than in those who had received the adjuvanted vaccine. This effect was even more pronounced in patients with respiratory and cardiovascular disease [21].

### Pneumococcal disease

Invasive pneumococcal disease (bacteremia, meningitis etc.) mainly affects young children and older adults [22, 23]. *S. pneumoniae* is also a common cause of community-acquired pneumonia (CAP) in the elderly [24]. A 23-valent polysaccharide vaccine has been used for many years for older adults, but polysaccharides induce IgM-dominated antibody responses without adequate immunological memory, as they are T cell-independent antigens. Conjugated vaccines have been developed for the vaccination of infants and very successfully reduced the burden of disease in children. A 13-valent conjugate vaccine has been introduced also for older adults. In a large randomized trial in persons over 65 years of age it was demonstrated that 45.6% (95.2% CI 21.8-62.5, *p* < 0.001) fewer first episodes of vaccine-type CAP requiring hospitalization and 75.0% (95% CI 41.4-90.8, *p* < 0.001) fewer first episodes of vaccine-type invasive pneumococcal disease occurred in the vaccine group compared to placebo [25]. Vaccination recommendations for *S. pneumoniae* are heterogeneous. Some countries still recommend the polysaccharide vaccine, while others recommend the conjugate vaccine alone or followed by the polysaccharide vaccine usually at least one year later. Details regarding the recommendations in European countries are published by the European Centre of Disease Prevention and Control. With the introduction of a 7-valent and a 10-valent conjugate vaccine for childhood vaccination around the year 2000 disease incidence and carriage of the serotypes included in the vaccines decreased in children. Consequently, transmission of these serotypes to older adults and therefore disease incidence in the older age group also decreased in the following years. However, serotype replacement was observed, which means that the incidence of pneumococcal disease caused by other serotypes, not included in the conjugated vaccines, increased both in children and older adults [26–28]. Similar effects have been observed for the 13-valent vaccine [29–31].

### Herpes zoster

Almost all adults are latently infected with varicella zoster virus (VZV). The primary infection, which usually occurs in childhood, manifests as chickenpox and live-long latency is established afterwards. Partial reactivation of the virus probably occurs frequently throughout life, but is usually controlled by virus-specific T cell responses. In the absence of sufficient immunological control, e.g. due to immunosuppression or immunosenescence, viral reactivation can lead to herpes zoster (shingles) [32]. The incidence of herpes zoster increases with age and it has been estimated that up to 50% of all cases affect persons older than 85 years [33, 34]. In a fraction of the patients acute episodes of herpes zoster are followed by post-herpetic neuralgia (PHN), characterized by long-lasting severe pain after the resolution of the zoster rash. The incidence of this complication is higher in older zoster patients, where it occurs in approximately one third of the cases [35]. Particularly in older patients, PHN frequently leads to substantial impairment in activities of daily living or even loss of independence [36, 37]. A single-shot immunization with an attenuated live-vaccine against herpes zoster, which has been licensed for use in older adults in 2006, is recommended in some countries. This vaccine induces T cell and antibody responses [38]. It was shown to reduce the incidence of herpes zoster by 51.3% (95% CI 44.2-57.6) and the incidence of PHN by 66.5% (95% CI 44.5-79.2) compared to placebo in a large randomized trial including persons older than 60 years [39]. The protective effect of the vaccine was lower in the very old, and long-term follow-up studies showed that protection waned over time, dropping to 21.1% (95% CI 20.9-30.4) for the prevention of herpes zoster and 35.4% (95% CI 8.8-55.8%) for PHN in years 7-10 [40, 41]. Antibody responses to a second dose of the vaccine more than 10 years after the first dose were similar to the first response, but cellular immune responses were higher after the booster dose. These findings indicate that there was a residual effect of the first vaccination on cellular immunity more than 10 years later and that the second dose induced a booster response [42]. Repeated vaccination of older individuals at appropriate intervals could therefore be considered for future recommendations.

## Vaccines recommended for all adults

Regular booster vaccinations against tetanus and diphtheria, in some cases combined with pertussis and/or polio, are recommended in many countries for all adults, including the elderly (summarized in Table 1). Regular vaccination against other pathogens is recommended in some countries, e.g. against tick-borne encephalitis in endemic areas. Most countries do not have specific recommendations for older adults, but e.g. in Austria, France, Liechtenstein and Portugal booster intervals are shortened for persons over 65 years.

### Tetanus and diphtheria

Tetanus- and diphtheria-specific antibody concentrations are frequently below the levels considered to be protective for adults, and are even lower for the elderly [43–48]. We could show that approximately 10% of a healthy elderly cohort recruited in Austria did not develop protective antibodies against diphtheria after a single booster shot and that almost half of the participants did not have antibodies above protective levels 5 years later. A second booster shot at this time point did again not provide long-term protection [48, 49]. This could be due to insufficient priming earlier in life, inadequate boosters throughout adulthood or age-related defects of the immune system. Given the fact that tetanus-specific antibody concentrations were substantially higher in the same cohort, one could also speculate that the currently used combination vaccine, which contains a reduced amount of diphtheria toxoid, might not be optimal. A more detailed overview of tetanus and diphtheria vaccination of the elderly has recently been published [50]. Antibody responses to booster vaccination against tick-borne encephalitis are also lower in old compared to young adults [51, 52].

### Pertussis

Vaccination against pertussis is widely used and accepted in the pediatric setting, but epidemiological data show an increased incidence of pertussis in adults and particularly in the elderly, for whom the infection can be associated with severe symptoms and increased mortality [53–55]. Adults can also transmit the disease to newborn infants, who are too young to be vaccinated. Few countries recommend regular booster immunizations with combined vaccines containing tetanus, diphtheria and pertussis antigens, whereas some recommend only one dose of pertussis-containing vaccine during adulthood. Many countries, however, do not recommend booster vaccination against pertussis for adults. Booster doses of combined tetanus/diphtheria/pertussis vaccine are well tolerated and immunogenic when given periodically to young or older adults [56], but antibody concentrations 4 weeks after vaccination are lower in old compared to young adults [46].

Appropriate vaccination documentation is crucial to deliver booster vaccinations at the right time points. Unfortunately, this documentation is often fragmentary for older adults, and it is therefore difficult to reliably assess adequate primary vaccination in childhood and the number of booster immunizations received throughout life. Several studies on tetanus/diphtheria vaccination reported only incomplete immunization histories [45, 47, 48]. Launay et al. reported that the number of vaccine doses received in life decreases from 7.1 (95% CI 6.9-7.2) doses of tetanus vaccine in young adults, which corresponds well with recommendations of 5 doses during childhood/adolescence and 10 year-booster intervals afterwards, to only 5.7 (95% CI 4.6-6.8) doses for adults aged 50-60 years [47]. This indicates a lack of regular booster immunizations during adulthood for this age group. Vaccination strategies for the future should include regular and well-documented booster shots throughout life, as post-booster antibody concentrations correlate with pre-booster antibody concentrations [46].

### Travel vaccines

Travel vaccines are becoming more important for older adults, as the number of older long-distance travelers increases due to improved health and mobility of this age group. Incidence and severity of typhoid fever and Japanese encephalitis are higher in older compared to younger adults [57, 58], highlighting the importance of travel vaccines for this age group. Vaccines against typhoid fever, Japanese encephalitis, rabies or yellow fever are neo-antigens for most older travelers and many older adults probably also never had contact with Hepatitis A and Hepatitis B. One hallmark of immunosenescence is the loss of naïve T cells and to a lesser extent of naïve B cells, which affects primary immune responses to these antigens. Impaired memory generation late in life has been demonstrated in animal models [59, 60] and therefore the success of primary vaccination late in life might be limited. Unfortunately, very few data are available regarding immune responses of the elderly to travel vaccines as most studies exclude older participants. Therefore, immunization guidelines rely primarily on studies with young adults. Antibody responses to Hepatitis A and B vaccines are already reduced in middle-aged adults compared to younger age groups [61, 62] and the percentage of non-responders without protective antibody concentrations against Hepatitis B increases with age [63]. Vaccination against Hepatitis B is not only relevant for older travels, but also for other risk groups, such as health care workers, household contacts of infected persons and hemodialysis patients, which also include older persons. Vaccination against yellow fever is mandatory for travelers to some South American and African countries. The live-attenuated yellow fever vaccine is highly immunogenic, but meta-analysis indicates that older adults have a higher risk for rare severe adverse events, such as e.g. yellow fever vaccine-associated viscerotropic disease, which mimics infection with the wild-type virus and has a high mortality of up to 60% [64].

## New vaccines developed for the elderly

### Next-generation vaccines against influenza, pneumococcal disease and herpes zoster

The current vaccines against influenza, *S. pneumoniae* and herpes zoster have several limitations. Vaccination against influenza has to be administered annually, as the vaccine provides protection only against the strains included in the formulation or closely related variants. Influenza virus strains are highly variable and new distinct strains circulate almost every year. A “universal” influenza vaccine inducing long-lasting immunity against all strains of influenza would solve the problem of annual re-vaccination and would probably improve compliance and vaccination coverage. Numerous candidate vaccines are investigated, which utilize various antigens, such as conserved regions of the surface proteins hemagglutinin and neuraminidase or internal viral proteins. Viral vectors, virus-like particles, DNA vaccines and the use of various adjuvants are discussed and tested as vaccine platforms in order to achieve potent CD4^+^ and CD8^+^ T cell responses, which are believed to be required for broad protection in addition to antibodies [65].

Universal pneumococcal vaccines would also be very useful, as there are approximately 100 serotypes of *S. pneumoniae*. Currently, vaccine manufacturers try to increase the number of serotypes included in conjugated vaccines, but antibody responses to polysaccharides will probably always be serotype-specific. Several pneumococcal proteins have been identified as potential vaccine candidates. They are highly conserved in all clinically relevant serotypes, and elicit potent immune responses in animal models. Additionally, whole-cell inactivated vaccines, live-attenuated vaccines and combinations of protein and polysaccharide components are investigated [66].

The incidence of herpes zoster is high not only in the elderly, but also in immunocompromised patients, such as after organ or stem cell transplantation, in HIV-positive individuals and in cancer patients, due a decline of cellular immunity. The current vaccine against herpes zoster contains live-attenuated virus and can therefore not be used for these patients due to safety issues [67, 68]. As described above, the efficacy of the vaccine is lower in the very old and wanes over time. Therefore, it is difficult to determine the optimal age for the vaccination, which is also reflected by differing age recommendations in the countries recommending the vaccine. Phase I/II studies demonstrated both the immunogenicity and safety of a novel inactivated vaccine containing the viral glycoprotein E in combination with the liposome-based AS01B adjuvant system (MPL and QS21) in older adults [69, 70] as well as in hematopoietic cell transplant recipients [71] and HIV-patients [72]. Large phase III randomized placebo-controlled trials demonstrated an overall clinical efficacy against herpes zoster of more than 90% with narrow confidence intervals. Remarkably, the vaccine efficacy was similar in all age groups, even in persons older than 80 years [73, 74]. This vaccine has recently been licensed in Canada and the US and might replace the live-attenuated herpes zoster vaccine in the future.

### New targets for vaccine development

We still lack vaccines for many pathogens that are of clinical relevance in the elderly. Respiratory syncytial virus (RSV) is a major cause of severe respiratory infection in infants, but usually causes only mild or moderate symptoms in adults. However, older and particularly frail individuals and patients with underlying co-morbidities can experience severe RSV disease. In the UK, an estimated 18,000 hospitalizations and 8400 deaths per year – almost exclusively in the elderly - are caused by RSV and underlying co-morbidity further increases the risk of severe disease [75]. Research on RSV vaccines was slowed down in the past, because a first-generation vaccine used in the 1960s for vaccination of infants was associated with risk for disease enhancement. Several candidate vaccines against RSV based on recombinant proteins, virus-like particles, live-attenuated virus or viral vectors are now in clinical development. It is crucial that these novel vaccines are not only developed for the pediatric market, but also include adult and elderly target groups.

There are many more infections affecting the elderly and various vaccine candidates are in pre-clinical or clinical development. One group of pathogens, which attracted special attention over the last years are nosocomial infections. The risk for these infections is high in the elderly, due to more frequent hospitalization and invasive procedures in this age group. Vaccines against bacterial nosocomial infections are highly desirable, as antibiotic resistance is a growing problem, but no such vaccine is currently on the market. The most severe infections are caused by *Clostridium difficile*, which is the most common cause of nosocomial diarrhea and *Staphylococcus aureus*, which is responsible for infections of prostheses, catheters or surgical wounds. Two vaccine candidates against *S. aureus* have been clinically tested, but unfortunately did not provide protection [76, 77]. Novel vaccine candidates are now in early stages of development. Most vaccine candidates against *C. difficile* are based on bacterial toxins which are responsible for the clinical symptoms [78]. Vaccines against these and other nosocomial pathogens, such as *Klebsiella pneumoniae*, *Escherichia coli* and the fungal pathogen *Candida spp*. have the potential to substantially reduce healthcare costs and to save many lives [79].

## Conclusion

Older adults are at high risk for infectious diseases and vaccination is an important preventive measure to facilitate healthy aging. Childhood vaccination programs are well-accepted and widely used, but unfortunately awareness for adult vaccination is by far less prominent. Several vaccines against influenza, *S. pneumoniae* and herpes zoster are available for the elderly and vaccines that are used throughout adulthood, such as tetanus, diphtheria and pertussis, are also relevant for the elderly. The first step towards optimal protection of the elderly is the comprehensive use of existing vaccines. Vaccination recommendations for adults and the elderly differ from country to country. Taking regional differences such as e.g. epidemiological parameters into account is of course necessary for optimal vaccine recommendations, but the diversity of recommendations e.g. throughout Europe can be confusing and might be interpreted as uncertainty. Therefore, increased efforts of harmonization would be desirable [80–82]. Nevertheless, without implementation strategies and sufficient vaccination coverage such guidelines provide only theoretical benefits. Vaccination coverage differs greatly between countries, and data are very difficult to obtain, as many countries do not have centralized databases collecting this information. The WHO goal of 75% influenza vaccination coverage for older adults (> 65 years) until 2014/2015 was not reached by most countries. The UK and the Netherlands reported the highest vaccination rates in Europe (above 70%), but some other European countries did not even reach 10% coverage in this age group [83]. Vaccine uptake for other adult vaccinations is not sufficiently documented in most countries and e.g. data on adult vaccination coverage against tetanus and diphtheria is only available for 5 of 29 European countries [84]. Improved vaccines against influenza, pneumococcal disease and herpes zoster have been developed over the last years and continuous effort is put into further increasing their potential to provide broad and long-lasting protection. In addition, vaccines against additional pathogens such as RSV and nosocomial infections could substantially improve health in old age. For the rational design of optimized and novel vaccines for the elderly basic research to understand immunosenescence is essential, and it is of utmost importance that clinical development and testing includes also older age groups.

## Abbreviations

*C. difficile*: *Clostridium difficile*
*Candida spp.*: *Candida species*
CAP: Community-acquired pneumonia
CI: Confidence interval
H: Hemagglutinin
HAI: Hemagglutination inhibition assay
IgM: Immunoglobulin M
ILI: Influenza-like illness
MPL: Monophosphoryl Lipid A
N: Neuraminidase
PCV: Pneumococcal conjugate vaccine
PHN: Post-herpetic neuralgia
PPV: Pneumococcal polysaccharide vaccine
RSV: Respiratory syncytial virus
*S. aureus*: *Staphylococcus aureus*
*S. pneumoniae*: *Streptococcus pneumoniae*
VZV: Varicella zoster virus
WHO: World Health Organization
